# Cardiac Amyloidosis and Valvular Heart Disease

**DOI:** 10.3390/jcm13010221

**Published:** 2023-12-30

**Authors:** Franz Duca, Christina Kronberger, Robin Willixhofer, Philipp E. Bartko, Jutta Bergler-Klein, Christian Nitsche

**Affiliations:** Department of Internal Medicine II, Medical University of Vienna, 1090 Vienna, Austria; franz.duca@meduniwien.ac.at (F.D.); christina.kronberger@meduniwien.ac.at (C.K.); robin.willixhofer@meduniwien.ac.at (R.W.); philipp.bartko@meduniwien.ac.at (P.E.B.); jutta.bergler-klein@meduniwien.ac.at (J.B.-K.)

**Keywords:** amyloid, aortic stenosis, mitral regurgitation, tricuspid regurgitation, ATTR, AL

## Abstract

Growing interest has accrued in the co-existence of cardiac amyloidosis and valvular heart disease. Amyloid infiltration from either transthyretin (ATTR) or of light chain (AL) origin may affect any structure of the heart, including the valves. The recent literature has mainly focused on aortic stenosis and cardiac amyloidosis, improving our understanding of the epidemiology, diagnosis, treatment and prognosis of this dual pathology. Despite being of high clinical relevance, data on mitral/tricuspid regurgitation and cardiac amyloidosis are rather scarce and mostly limited to case reports and small cases series. It is the aim of this review article to summarize the current evidence of concomitant valvular heart disease and cardiac amyloidosis by including studies on epidemiology, diagnostic approaches, screening possibilities, therapeutic management, and prognostic implications.

## 1. Introduction

**Figure A1:**
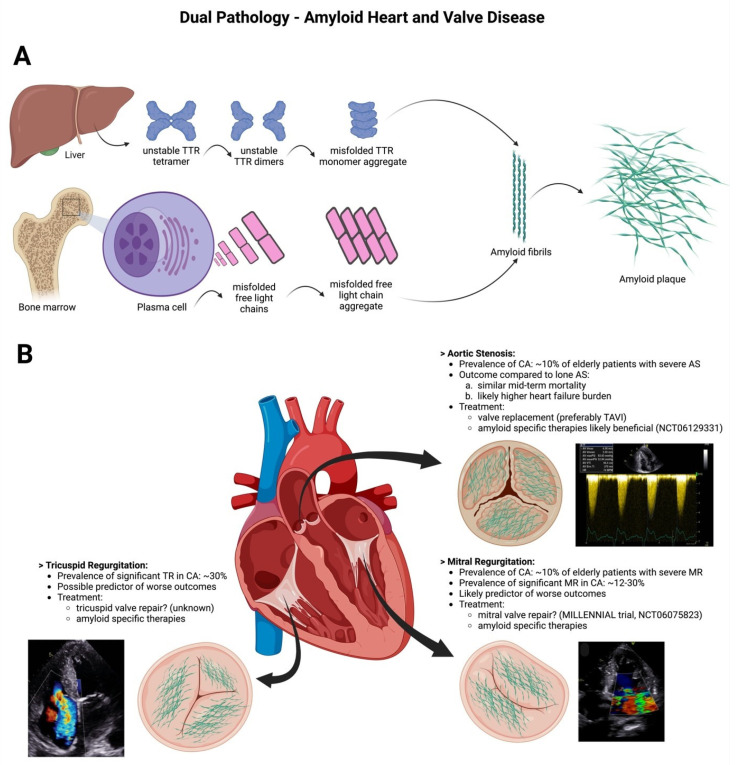
Part (**A**): The pathophysiology of myocardial and valvular amyloidosis (CA) involves amyloid deposition primarily attributed to transthyretin (ATTR) or light chains (AL) amyloid. Transthyretin, predominantly synthesized by the liver (95%), forms a tetramer that undergoes destabilization due to aging or point mutations in the TTR gene, leading to its dissociation into di- and eventually monomers. Subsequently, these monomers aggregate and form amyloid fibrils and plaques. In AL amyloidosis, a plasma cell clone produces misfolded free light chains that also aggregate and are deposited within the extracellular space of the myocardium. Part (**B**): Amyloid depositions are detected not only in the myocardium but also within valve leaflets and the valvular apparatus. These depositions are primarily associated with aortic stenosis, mitral regurgitation, and tricuspid regurgitation. Conversely, occurrences of aortic regurgitation, or mitral/tricuspid stenosis are exceedingly rare among patients with CA.

Cardiac amyloidosis (CA) is an infiltrative myocardial disease that causes heart failure and death via the deposition of amyloid fibrils. The two predominant amyloid proteins deposited in the heart are transthyretin (ATTR) and immunoglobulin light chain (AL). Formerly believed to be a rare condition, recent diagnostic advances and enhanced disease awareness have resulted in a significant increase in the number of patients diagnosed with CA [1] (Central Illustration—Figure A1A). The diagnosis of ATTR-CA has been facilitated by the introduction of a non-invasive diagnostic algorithm, which allows a non-biopsy diagnosis in the majority of patients with suspected CA [2]. The increased diagnosis of CA has spurred the in-depth characterization of this condition, which, among other things, has unveiled a frequent co-existence with significant valvular heart disease (VHD). VHD may impact the clinical manifestation, therapeutic management, and prognosis of patients with CA. While many issues have been addressed by recent research, there still exist essential gaps in knowledge that warrant investigation via prospective trials.

## 2. Methods

### 2.1. Aim of the Review

This research article aimed to (a) summarize our current understanding of concomitant CA and the most frequent forms of VHD, namely aortic stenosis, mitral regurgitation, tricuspid regurgitation, mitral stenosis, and tricuspid stenosis, and to (b) highlight gaps in knowledge that could stimulate the conductance of prospective studies.

### 2.2. Structure of the Review

The present review was structured to discuss and provide up-to-date knowledge on the epidemiology, pathophysiological considerations, screening possibilities, prognosis, and management options in patients with the dual pathology of CA and VHD.

### 2.3. Conduction of Literature Review and Study Appraisal

For this review, a comprehensive literature review in PubMed/Medline (updated October 2023) was performed using the following terms: amyloidosis, cardiac amyloidosis, valve, valvular, stenosis, and regurgitation.

Given that the design of this work was a narrative review and the general lack of large-scale, prospective, multi-center, randomized trials involving patients with the dual pathology of CA and VHD, no formal criteria for study selection or appraisal were enforced. The study methodologies are given in the reference section.

## 3. Aortic Stenosis

### 3.1. Epidemiology

The coexistence of CA and aortic stenosis (AS) was described for the first time in prospective patient populations in 2016 and has since then garnered rapidly growing interest [3,4]. These two preliminary studies found a prevalence of 12% (in elderly patients with a median age of 85 years) and 6% (in patients undergoing surgical aortic valve replacement and receiving intraprocedural biopsy with a mean age of 75 years), respectively. Another study by Castano et al. on elderly AS patients undergoing transcatheter aortic valve replacement (TAVR) even described a prevalence of 16%, which may have been a slight overestimation since the patients were not recruited consecutively [5]. Further studies with prospective consecutive screening followed, reporting on disease prevalences ranging from 8 to 13% [6,7,8]. However, one needs to consider the different definitions of CA in these studies. While CA was defined strictly according to the current expert consensus algorithm (Figure A3) [2] in the studies by Nitsche et al. and Dobner et al., the patients found to have AS–CA in the study by Scully et al. also included those with low-grade myocardial tracer uptake using bone scintigraphy and confirmed by SPECT/CT (Perugini grade 1). The largest multi-center, multi-cohort study to this day reported a prevalence of 12% [9]. Here, patients with grade 1 myocardial uptake (confirmation by SPECT/CT), which is believed to represent an early disease stage of ATTR, were included [10]. One recent meta-analysis summarized the current evidence gathered from screening studies and yielded a prevalence of 11% [11]. Among AS patients with a low-flow, low-gradient phenotype, the prevalence of concomitant CA is believed to be even higher, but no study has yet evaluated this in a population restricted to low-flow low-gradient AS.

To put the disease prevalence into perspective, the prevalence of CA in the general population needs to be considered. Respective estimates are derived from bone scintigraphy referrals for non-cardiac indications, which is currently considered the most accurate way to assess ATTR prevalence in the general population. The largest comparable study to this day reported the presence of intense cardiac tracer uptake (Perugini grade ≥ 2) in ~3% of consecutive patients aged 80 years or older (comparable to the age of patients in TAVR screening studies) [12]. Hence, the prevalence of CA is ~4 times higher in patients with AS compared to the “general population”. This warrants a discussion of the possible underlying pathophysiological mechanisms.

### 3.2. Pathophysiological Aspects

The underlying pathophysiological principles of dual AS–CA are currently incompletely understood and are limited to hypotheses. The “chicken or egg” discussion in AS–CA refers to the question of whether (a) the accumulation of amyloid fibrils in the aortic valve tissue is a driver of the progression of AS or whether (b) AS primes the left ventricle for amyloid deposition. Amyloid has indeed been identified in a high proportion of surgically resected heart valve specimens [13]. However, rather than common CA subtypes (AL and ATTR), ApoA1 was found, which aggregates in the atherosclerotic and inflammatory milieu of AS. Nevertheless, data regarding the contributing role of amyloid deposition in the disease progression of AS are conflicting, as one recent study reported a surprisingly high rate of 58% of ATTR deposition in aortic valve tissue [14]. A different hypothesis regarding the increased susceptibility of the left ventricular myocardium to amyloid formation and deposition is supported by the mechano-enzymatic cleavage process, which has been proposed previously [15]. Increased biomechanical forces in the setting of increased shear stress caused by elevated left ventricular afterload may initiate the proteolysis/fibrillogenesis pathway. Finally, both AS and CA share an endo- to epicardial gradient with regard to fibrosis formation and amyloid deposition, respectively [16]. It is plausible that the ischemic stress caused by AS creates a milieu that facilitates amyloid deposition, such as that described for amyloid ß in the brain [17]. The fact that the LV mass index has been described to be lower in AS-ATTR compared to matched lone ATTR cardiomyopathy (ATTR-CM) patients has also led to the hypothesis that AS–CA likely represents an early stage of amyloid infiltration [18].

### 3.3. Diagnosis and Screening

The diagnostic pathway of detecting concomitant CA in patients with AS does not differ from the conventional algorithm recommended by the current expert consensus documents and guidelines [2,19]. The difficulty lies in the enormous number of aortic valve replacements being performed in large valve clinics, which makes it logistically impossible to perform CA testing in every AS patient. Hence, efforts have been made to identify parameters that can be used to assess the pre-test probability of the presence of concomitant AS. Among the parameters reported to indicate a higher likelihood of co-existing CA are higher age, male sex, history of carpal tunnel syndrome, decreased stroke volume (low-flow pattern), poorer diastolic function, out-of-proportion LV thickening, the presence of a right-bundle branch block on ECG, the discordance of LV mass on ECG and echo (low “voltage/mass ratio”), and out-of-proportion troponin elevation [5,6,8,9,20]. Some of these measures have been combined to form an additive score (RAISE score) that includes proposed optimal cut-off values for continuous metrics [9]. Here, each parameter is assigned a specific score if present. Higher scores indicate a higher pre-test probability of concomitant CA, and patients with a score of 3 points or higher are advised to receive further testing using bone scintigraphy and light chain assessment. It is important to mention that this score has been developed in a patient population with severe AS referred for TAVR and should therefore be applied to this specific group only. It is currently unknown how the RAISE score would perform in other clinical scenarios, such as (a) patients with AS of moderate or mild severity, (b) patients with a history of AS who have already received an aortic prosthesis, or (c) patients without AS. AS patients may also be suspected of concomitant CA if red flags are present in other tests (e.g., polyneuropathy, deafness) or imaging modalities (e.g., characteristic pattern of late gadolinium enhancement or elevated T1 relaxation times/extracellular volume fraction on cardiac magnetic resonance imaging—Figure A1). Finally, patients awaiting TAVR require a cardiac computed tomography (CT) scan as part of their pre-procedural planning. Just like the contrast agents used in cardiac magnetic resonance, CT contrast agents also represent extracellular agents, not entering myocardial cells. This feature can be exploited to acquire and quantify the extracellular volume fraction using CT. Significantly higher CT-ECV values have been described for AS–CA compared to lone AS, and CT-ECV therefore represents a highly attractive marker for screening all AS patients undergoing TAVR work-up for concomitant CA [21].

### 3.4. Treatment and Outcomes

Given the high prevalence of comorbidities and advanced age in AS–CA, TAVR was previously thought to be futile [22]. These assumptions were also based on initial reports of higher mortality in AS–CA patients compared to lone AS [4,20]. However, these were preliminary studies that either investigated younger patients receiving surgical aortic valve replacement or selected patients, of whom only ~50% underwent aortic intervention. Further studies assessing the short-term mortality and hospital admissions for heart failure post TAVR followed [6,23]. Both of these studies reported a similar risk of mortality and heart failure hospitalization post intervention for dual AS–CA and lone AS. However, in the study by Rosenblum et al., a higher hospitalization rate (n per person years) within the first year after TAVR was found for AS–CA compared to lone AS [23]. A similar mortality for both groups up until 3 years post TAVR was confirmed by the largest cohort study to this day, and also by one recent study [7,9]. Importantly, the idea that the treatment of severe AS in patients with dual pathology was futile was disproven by the study of Nitsche et al., which demonstrated clear survival benefits for patients with AS–CA receiving TAVR compared to those with conservative care [9]. Besides valvular treatment, amyloid-specific therapies should also be discussed for AS–CA. The question of whether patients with AS–CA should receive amyloid-specific treatment on top of valve replacement currently represents a gap in the evidence, as patients with significant valve disease were excluded from large ATTR-CM drug trials [24,25]. Also, AS–CA cohort studies were underpowered to address this issue, and patients with AS–CA receiving ATTR-specific treatment were underrepresented [6,9]. A large international, multi-centre, multi-cohort registry is currently trying to address the potential benefit of ATTR-specific therapies in dual AS–CA (NCT06129331).

Despite the lack of convincing evidence, surrogate markers may suggest possible benefits through ATTR-specific treatment in AS–CA. One study reported the trajectories of functional capacity, laboratory makers, and echocardiographic parameters in AS–CA compared to lone AS at one year after TAVR [26]. Patients with AS–CA remained more symptomatic, with higher residual NT-proBNP values and a higher LV mass. Also, a pattern of apical sparing developed in AS–CA only after TAVR. These features indicate that, at one year after TAVR, AS–CA resembles a lone ATTR-CM phenotype by symptoms, biomarkers, morphology, and contractility pattern, making the benefits of ATTR-specific drugs very likely.

### 3.5. Potential Clinical Implications

Current evidence suggests that patients with severe AS should be screened for concomitant CA in case of increased pre-test probability (RAISE score). Severe AS in patients with dual pathology should be treated using valve replacement (preferentially TAVR) to improve prognosis. Additional amyloid-specific treatment on top of valve replacement is likely to be beneficial, but this remains to be proven (NCT06129331).

## 4. Mitral Regurgitation

### 4.1. Epidemiology

Significant (moderate or higher) mitral regurgitation (MR) can be found in approximately one third of all patients with heart failure (HF) [27]. However, its prevalence among patients with CA is much less established. The most recent study to specifically investigate MR in the context of CA included 877 patients from the United Kingdom. Chacko et al. reported a prevalence of 12.9% of at least moderate MR in patients with ATTR-CM [28]. Interestingly, an earlier study from the same UK cohort and first author found significant MR in 30.2% of their 1240 patients [29]. Moreover, data from other cohorts also suggest higher prevalences of 17% and 24%, respectively [30,31]. Conversely, studies investigating the prevalence of CA in patients with significant MR are scarce and heterogeneous in their design. One study by Donà and colleagues systematically screened patients undergoing transcatheter edge-to-edge mitral valve repair (TEER) for the presence of CA and found a dual pathology in as many as 19.2% (whereby 11.7% had definite CA, as defined according to the current guidelines) [32]. Another recent study from the Cleveland Clinic by Xu et al. took a different approach and histologically analyzed > 7700 surgically removed mitral valve specimens. Of note, amyloid was present in only 0.2% of their specimens [33]. In contrast, a German study found amyloid in 20.8% of mitral valve specimens. However, none of the common amyloid proteins (AL, ATTR) were identified by immunohistochemical analysis, and a major limitation was the small study size of only 24 MR patients [13].

Regarding the clinical characterization of individuals affected by CA and concurrent MR, Donà et al.’s work indicates that these patients tend to be older and more often male when compared to patients with lone MR [32]. However, large comparative studies in the context of CA and MR are lacking.

### 4.2. Pathophysiological Aspects

From an etiological standpoint, MR in CA features aspects of primary and secondary MR. On the one hand, amyloid depositions alter the structure and function of the valve leaflets and the (sub-)valvular apparatus itself [28,33]. A Japanese study from the year 2000 provides interesting insights into how amyloid infiltration changes the physical properties and subsequently the function of the mitral valve using an acoustic microscope [34]. The authors found that the mitral valves from CA patients with at least moderate MR were significantly stiffer than the valves from CA patients with only mild MR or the controls. Of note, more unexpected presentations of MR in CA, such as ruptured chordae, have also been published [35]. Moreover, CA is associated with distinct LV geometry changes, which in turn can also cause insufficient mitral valve closure [28,36,37]. Finally, left atrial enlargement represents a hallmark sign of restrictive cardiomyopathies, often leading to mitral valve annular dilatation and atrial functional MR [32]. Mixed etiologies may also occur. Thus, MR in the context of CA is indeed a rather complex disease, which is underlined by the fact that it can present as Carpentier classes I-III [38].

### 4.3. Diagnosis and Screening

Similar to AS in CA, the diagnostic algorithm for CA in the context of MR does not differ from current recommendations [2,19]. Given the relatively high prevalence of CA among patients undergoing TEER, one could argue that systematic screening should be implemented in order to avoid missing a diagnosis of underlying CA. Again, this approach is probably not executable, especially in high-volume valvular heart disease clinics where demands for bone scintigraphy would then vastly exceed the number of available slots.

Given the unique pathophysiology and hemodynamics present in patients with CA, the bigger challenge is the assessment and grading of MR itself. To date, despite being regularly used, it is not clear whether the recommended cut-offs for the quantification of MR in the context of CA should be applied [39]. It is likely that in the setting of low-flow conditions, the cut-point values of quantitative MR metrics (regurgitant volume, proximal isovelocity surface area, effective regurgitant orifice area) indicating poor clinical outcomes differ from those used in normal-flow conditions [40]. Thus, prospective studies are needed in order to establish valid cut-offs and guidelines for the quantification of MR in CA.

### 4.4. Treatment and Outcomes

Although numerous studies have investigated the prognostic power of MR and how it should best be treated in patients with heart failure and reduced ejection fraction, respective data are extremely scarce in CA. The results from a recent study of 877 ATTR-CM patients by Chacko et al. suggest that MR is indeed of prognostic relevance in this patient population. The authors found that an increase in MR severity, irrespective of the baseline severity, was among the strongest predictors of outcome in their cohort. A 2020 study by the same authors found that a MR ≥ moderate at baseline was associated with adverse outcomes only in their univariable survival analysis, but not after adjusting for the National Amyloidosis disease stage [29]. Therefore, more studies dedicated to the prognostic role of MR in CA are clearly needed.

Thus far, two studies have investigated the feasibility of TEER as a treatment option in this patient population [32,41]. One German study included five CA patients (four ATTR, one AL) who underwent TEER, which was demonstrated to be feasible, with a procedural success rate of 100% and a sustained reduction in MR severity [41]. The authors also proposed a potential benefit regarding survival in CA patients undergoing TEER when compared to a control group of CA patients with severe MR not receiving TEER. However, this statement seems oversized, as their control cohort consisted of a much sicker patient population (NTproBNP: 17,365 pg/mL versus 2928 pg/mL; eGFR: 28 mL/min versus 82 mL/min). The second study included 120 consecutive patients who underwent TEER at two Austrian university hospitals [32]. Among these, 23 patients had concomitant CA. Again, the procedural success rate was 100% and the MR severity could be significantly reduced. With respect to outcomes, patients with a dual pathology had a higher risk of heart failure hospitalizations, but similar mortality when compared to lone MR.

In summary, TEER appears to be technically feasible in patients with CA with a sustained reduction in MR severity. It is yet unclear whether TEER also improves the quality of life and prognosis of CA patients, as these patients were specifically excluded from large TEER trials (MITRA-FR, COAPT). A current randomized trial is trying to answer this question and is recruiting patients with ATTR-CM and significant MR; these patients are being randomized to receive either (a) TEER plus optimal medical therapy or (b) optimal medical therapy alone (MILLENNIAL trial, NCT06075823).

### 4.5. Potential Clinical Implications

Due to the limited amount of data and the relatively low level of evidence, conclusions about the clinical implications of and therapeutic strategies for concomitant MR and CA remain speculative. However, insights from studies conducted by Xu et al. and Donà et al. may offer some guidance. Their studies imply that regular screening for CA among individuals undergoing mitral valve surgery might not be necessary, whereas patients with MR planned for TEER should undergo further assessment for the presence of CA in case of increased pre-test risk. Regarding the therapeutic management of MR in the context of CA, no specific recommendations besides the initiation of amyloid-specific therapies can be given at this stage. Enrollment of dedicated clinical trial is currently ongoing.

## 5. Tricuspid Regurgitation

### 5.1. Epidemiology

Dedicated studies regarding the role of tricuspid regurgitation (TR) in patients with CA are scarce. A 2021 study by Fagot et al. from France assessed the prevalence of TR in 283 CA patients [31]. The authors reported a prevalence of TR graded as ≥ moderate of 26.2%, with only a slight difference when patients were stratified according to the amyloid subtype (AL: 23.4%, ATTR: 27.9%). This would suggest that the TR prevalence in CA is comparable to other heart failure entities. Interestingly, the study by Fagot and colleagues did not find differences with respect to clinical characteristics (e.g., age, gender, presence of concomitant MR) when comparing CA patients with and without significant TR [31]. However, large studies dedicated to the clinical characterization of patients with a dual pathology of CA and TR are missing.

### 5.2. Pathophysiological Aspects

Even though amyloid infiltration can be found in the tricuspid valve, the fact that a significant proportion of CA patients in the study by Fagot et al. exhibited tricuspid annulus dilatation suggests that the TR mechanism in CA might be similar to other forms of heart failure (mostly secondary to elevated pulmonary pressures) [28,31,42,43]. However, results from the UK study by Chacko and colleagues argue against this theory, as only 16% had tricuspid annulus dilatation and most patients presented with thickened and restricted valve leaflets [28].

### 5.3. Diagnosis and Screening

Clinicians should adhere to current recommendations and guidelines for the quantification and assessment of TR in the context of CA [39]. However, once again, additional data are needed to determine whether different cut-offs are needed in this particular patient population.

### 5.4. Treatment and Outcomes

The debate of whether TR is an independent predictor of adverse clinical outcomes and whether it represents a valid therapeutic target spans over decades [44,45]. Although much less data is available for patients with CA, this data is also conflicting [28,31]. In the studies by Chacko and co-authors, a deterioration in MR but not TR was a strong predictor of adverse outcomes [28,29]. Conversely, in the study by Fagot et al., the presence of ≥ moderate TR was associated with increased mortality. Nonetheless, this association was found only in patients with ATTR but not AL–CA [31].

Given the general frailty and advanced age of patients with CA, a minimally invasive treatment approach seems preferrable to an open-heart surgery. The largest case series thus far was published by the Amyloidosis Center in Heidelberg. In total, 8 ATTR-CM and 21 non-CA patients underwent transcatheter tricuspid valve repair with the PASCAL Ace System [46]. The procedural success rate was 100%, and after three months of follow-up, the TR severity and NYHA class were substantially improved. The CA patients were furthermore compared to a non-matched control group of patients with severe TR and no CA, and the authors did not find any differences with respect to outcome endpoints (all-cause death/heart failure hospitalization). Randomized trials are needed to evaluate whether TR repair improves clinical outcomes in patients with CA.

### 5.5. Potential Clinical Implications

**Figure A2:**
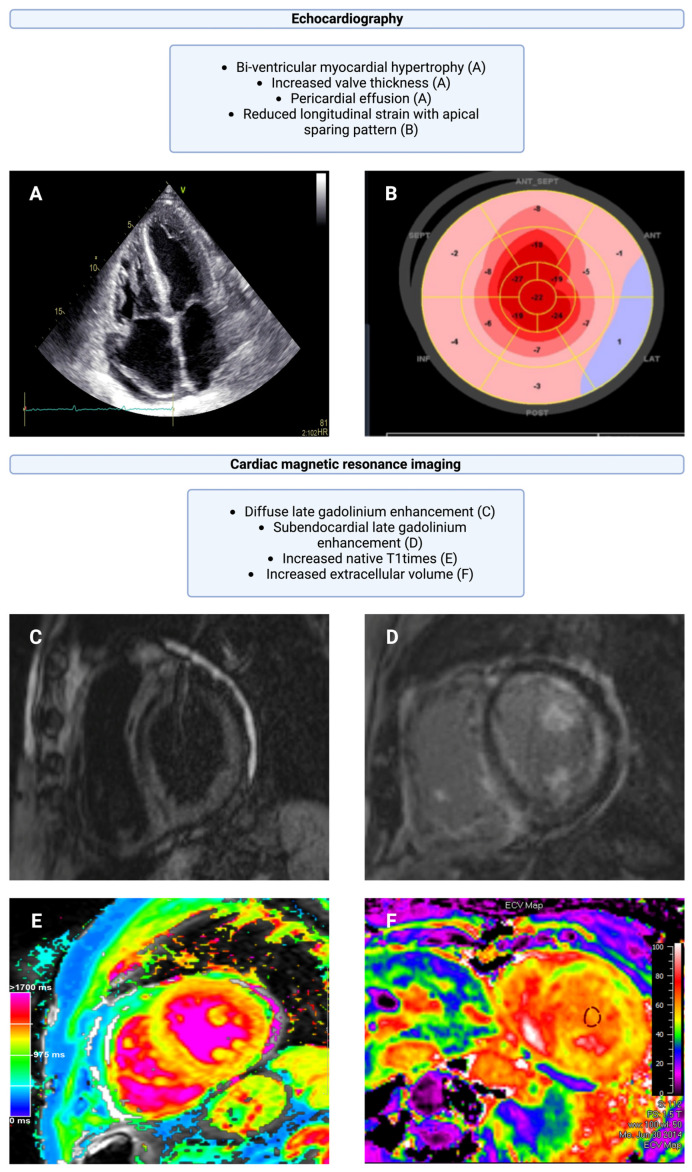
Cardiac amyloidosis “red flags” in multimodality imaging. Several echocardiographic and cardiac magnetic resonance imaging “red flags” should raise the suspicion for cardiac amyloidosis. These include bi-ventricular myocardial hypertrophy (Panel (**A**)), increased valve thickness (Panel (**A**)), pericardial effusion (Panel (**A**)), reduced global longitudinal strain with apical sparing (Panel (**B**)), diffuse late gadolinium enhancement [(LGE), Panel (**C**)], ring-like subendocardial (up to transmural) LGE (Panel (**D**)), increased native T1 times (Panel (**E**)), and increased extracellular volume (Panel (**F**)).

**Figure A3:**
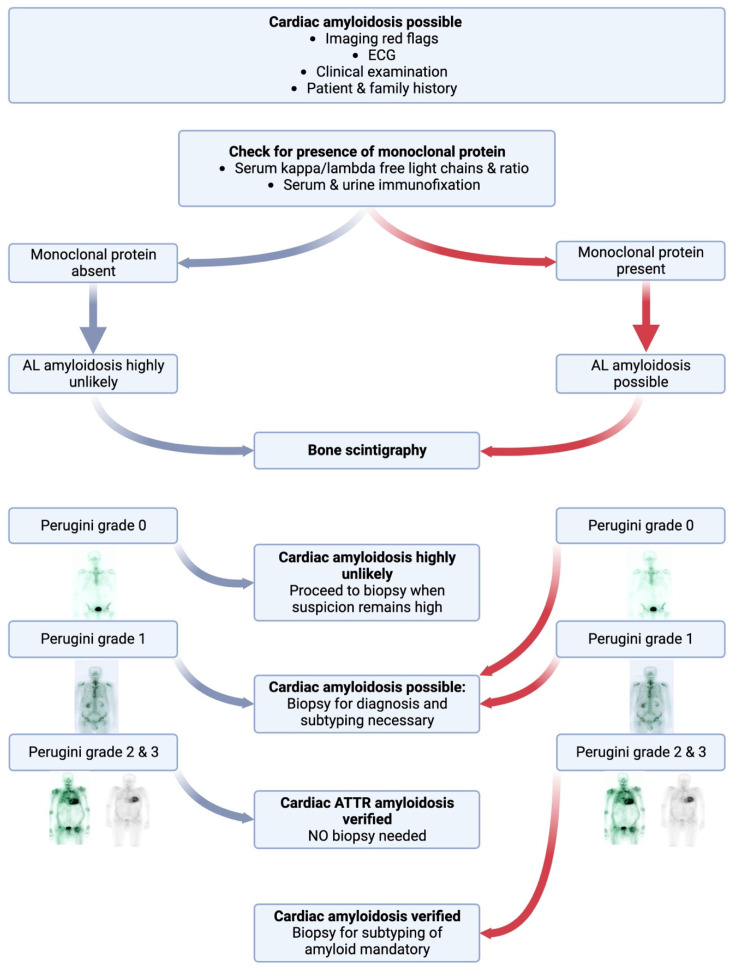
Diagnostic pathways in cardiac amyloidosis. Patients in whom cardiac amyloidosis is suspected due to clinical examination, clinical history, ECG, or imaging red-flags (Figure A2) should be tested for the presence of a monoclonal protein and should undergo bone scintigraphy. In the absence of a monoclonal protein and positive bone scintigraphy (Perugini grade ≥ 2) cardiac transthyretin amyloidosis (ATTR) can be diagnosed without the need for biopsy. When bone scintigraphy is negative (Perugini grade 0) and no paraprotein is detected, CA is highly unlikely and other differential diagnosis should be considered. In patients with other diagnostic combinations biopsy is mandatory.

Compared to other forms of VHD in CA, the lack of data is particularly evident in TR, making it even more difficult to draw conclusions for clinical practice. Like with other forms of (significant) VHD, the screening of elderly patients with TR should be sought in the case of additional red flags (Figure A2). Whether TR in the context of CA should be specifically targeted by intervention needs to be addressed by a randomized controlled trial.

## 6. Mitral and Tricuspid Stenosis

Due to the infiltrative nature of CA, one could hypothesize that amyloid build-up within the mitral and tricuspid valve leaflets should frequently lead to the stenosis of these valves. However, studies from CA expert centers with large and well-characterized patient cohorts have not found atrioventricular (AV) valve stenosis to be of clinical relevance [29,30]. This very low prevalence is also supported by a study from Xu et al., in which possibly amyloid-related mitral valve stenosis was found in only 2 out of 7733 histologically assessed mitral valve specimens. Furthermore, our literature review yielded only two case reports on concomitant AV valve stenosis in CA (one AL and one Serum amyloid A amyloidosis) [47,48]. This low prevalence may partly be explained by the AV annulus dilatation, which is commonly observed in CA and counteracts possible stenotic processes [37].

## 7. Gaps in Knowledge and Recommendations for Future Research

This section summarizes the current gaps in the knowledge, partly already mentioned in the previous sections of this manuscript.

### 7.1. Aortic Stenosis

The long-term clinical outcomes in AS–CA compared to lone AS after valve replacement are currently unknown. In particular, the potential differences in the outcomes between AS–CA patients with “early” infiltration (Perugini grade-1 uptake) and “advanced” ATTR-CM warrant investigation. Similarly, the treatment effect of amyloid-specific medication on top of valve replacement remains to be explored. Results from a large-scale prospective international registry dedicated to this research question are expected in 2024 (NCT06129331). Finally, the pathophysiological aspects underlying the higher prevalence of CA among patients with AS compared to the general population are not fully known.

### 7.2. Mitral and Tricuspid Regurgitation

Knowledge gaps regarding MR/TR in CA concern not only their epidemiology, but also the applicability of current MR/TR grading criteria in this hemodynamically distinct patient population. Additionally, it remains uncertain whether significant MR/TR are indeed independent predictors of worse outcomes and could therefore serve as potential therapeutic targets.

Future research endeavors focused on MR/TR in CA should concentrate on several key areas: (a) determining the prevalence of MR/TR in CA, (b) establishing the prevalence of CA in patients presenting with MR/TR, (c) assessing the prognostic implications of concomitant MR/TR in the setting of CA, and (d) evaluating the therapeutic potential of MR/TR repair, particularly in light of effective amyloid-specific pharmacotherapies.

Finally, collaborations among dedicated amyloidosis centers seem crucial to facilitating the conductance of large-scale cohort studies and randomized trials.

### 7.3. Considerations in Trial Design for VHD in CA

Due to the increased disease awareness and advancements in diagnostic modalities, CA is now commonly diagnosed at earlier stages. Consequently, when calculated from the time of diagnosis, patients exhibit lower event rates—both in the absence of amyloid-specific medication and even more so if under treatment. As an illustration, event rates in the placebo arm of the early ATTR-ACT trial were much higher compared to those of the recent ATTRibute-CM trial. These developments should be considered when designing clinical trials assessing the benefits of amyloid therapies. Surrogate endpoints reflecting disease severity should be included as primary study endpoints. The selection of hierarchical endpoints that value hard clinical outcomes the most, followed by surrogate endpoints (e.g., Finkelstein–Schoenfeld method), represents one opportunity to decrease the required trial size (e.g., NCT06075823). Such surrogate parameters in CA include the six-minute walk distance, the NYHA functional class, NT-proBNP trajectories, quality of life, as assessed using dedicated questionnaires, and the extracellular volume fraction by CMR, among others. Furthermore, enhancing communication between healthcare professionals and patient advocacy groups holds potential in identifying appropriate outcome parameters in this difficult-to-treat patient population.

## 8. Conclusions

In summary, valve disease and CA frequently coexist. While the dual pathology of AS and CA has been studied quite extensively, thereby closing significant gaps in knowledge, the coexistence of mitral/tricuspid regurgitation with CA is less well investigated (Central Illustration—Figure A1B). Modern imaging techniques enable non-invasive diagnosis in a large proportion of patients with suspected CA. CA screening should therefore be widely applied in order to not miss a diagnosis of concomitant CA in patients with significant valve disease. Minimally invasive and transcatheter techniques seem most appropriate when aiming to treat valve disease in this elderly patient population with a high burden of comorbidities. This has been demonstrated for elderly patients with dual AS–CA. The treatment effects and associated prognosis of edge-to-edge repair in patients with mitral/tricuspid regurgitation and CA are yet to be determined [44,45].
